# How We Perceive Others Resembling Us

**DOI:** 10.1177/2041669520966623

**Published:** 2020-11-25

**Authors:** Alexandra Hoffmann, Thomas Maran, Pierre Sachse

**Affiliations:** Department of Psychology, 27255 University of Innsbruck, Innsbruck, Austria; Department of Entrepreneurship, University of Liechtenstein, Vaduz, Liechtenstein; Department of Psychology, 27255 University of Innsbruck, Innsbruck, Austria

**Keywords:** self-face, similarity, familiarity, eye-tracking, morphing, face perception

## Abstract

Eye contact is essential for social cognition, acting as an important tool for social communication. While differences in face scanning patterns concerning familiarity have been thoroughly investigated, the impact of facial similarity on gaze behavior has not been examined yet. We addressed this topic by recording subjects’ eye-directed gazing while looking at faces that were individually created systematically varying in terms of similarity to the self-face and familiarity. Subjects’ self-faces were morphed into three other faces including a close friend of the same sex. Afterwards, they rated similarity to their self-face of those morphed face stimuli in a separate rating task. Our results show a general preference for the eyes’ area as well as differences regarding fixation patterns depending on similarity to the self-face. The lower the similarity to the self-face, the more fixations on the eyes’ area. Subjects’ ratings followed a linear line, indicating well-pronounced face perception. Nevertheless, other faces were rated faster than the self-face independent of familiarity, while morphed faces got the slowest ratings. Our results mirror the importance of similarity to the self-face as a factor shaping the way we look at the eyes of others explaining variance apart from familiarity.

Imagine yourself walking through a shopping mall when being confronted with a completely unfamiliar person in front of you. Your first reaction would be trying to capture their intentions. Is she or he looking at me? Is there something wrong? To do so—research findings suggest—you would focus intensely on the eyes of this individual (Baron-Cohen, 1997). The stranger someone seems to us, the more we feel the need to fathom what he or she is up to (DeBruine, 2002). However, what would happen if this individual highly resembled us? Studies investigating eye movement patterns ascribe a special role to the self-face (e.g., Devue et al., 2009; Li & Tottenham, 2011). But although evidence suggests our self-face to influence face perception and recognition, no study to date investigated how facial resemblance shapes the way we look at other faces. Thus, our study aims to do exactly this. The purpose of this article is the investigation of how similarity to the self-face influences our attention toward the eyes of other individuals who are either unfamiliar or familiar to us. We did so by confronting individuals with faces manipulated to resemble their own face to a gradually changing degree, while tracking their gaze patterns. We focused on eye-directed gazing, as convincing evidence supports its importance for both social information gathering as well as signaling processes in social encounters (Risko et al., 2016). More specifically, unfamiliar and dissimilar individuals are screened more closely than familiar or similar ones, as they are perceived as less trustworthy (Cassidy et al., 2017; DeBruine, 2002; DeBruine et al., 2008). Thus, a first response to reduce ambiguity about the intentions of a social encounter is to put attention toward their eye region, which allows reliably inferring others’ mental states (Baron-Cohen, 1997; Kobayashi & Kohshima, 1997).

## Theoretical Background

The self-face plays a special role when investigating face perception, as our self-face representation is strongly related to the ability to recognize facial expressions and emotions (Finke et al., 2017; Li & Tottenham, 2011). When being primed with the self-face, facial expression processing as well as visual exploration of faces are enhanced (Li & Tottenham, 2011). Similarity to the self-face also leads to deeper processing of emotional faces, particularly in happy faces (Finke et al., 2017). Moreover, when searching for a face within a crowd, our own face strongly interferes with the searching, as it is rather difficult to avoid and seems to draw more attention than other faces. This pattern was not only found for the self-face but also for other familiar faces, thus indicating that familiarity might play another important role (Devue et al., 2009). When investigating differences in face-scanning strategies between unfamiliar faces and the self-face, no differences were obtained although a self-face advantage both in healthy and prosopagnosia subjects was shown (Malaspina et al., 2018). Subjects had to decide whether a chimeric face represented their own face or another unfamiliar face. Although these results speak for a special processing of the self-face, eye movement patterns during perception of faces similar to the self-face have never been investigated.

However, not only behavioral but also neural results speak for a special processing of the self-face. Activation of face-preferential regions increases with visual information, independent of familiarity. Medial temporal lobe structures as well as the anterior inferior temporal cortex are activated after information accumulation for face familiarity (Ramon et al., 2015). Uddin et al. (2005) found a unique network to be involved in self-face recognition, which comprised of frontoparietal structures that are part of the mirror neuron system, while for familiar faces no special activation was found. Platek and Kemp (2009) confirmed this difference between familiar and self-face, but highlighting that differences are subtle. Earlier electroencephalography (EEG) studies found the self-face to produce special electrophysiological responses similar to newly learned familiar faces (Tanaka et al., 2006). Recent research investigating identity-specific neural responses with EEG (Campbell et al., 2020) found a larger bilateral response in the occipital–temporal region to the self-face compared with familiar and unfamiliar faces indicating an increased electro-physiological response for the self-face during face recognition, though. Increased event-related potentials were furthermore found when comparing the self-face with familiar and unfamiliar faces, pointing toward a specialty of the self-face (Keyes et al., 2010).

Familiarity also influences our gaze behavior in a specific way, supporting a functional view on how we perceive strangers or more specifically their eyes. For example, the eye region is fixated more often in unfamiliar than familiar faces (Althoff & Cohen, 1999; Barton et al., 2006; Heisz & Shore, 2008). Numerous studies have shown that recognition of familiar faces is faster and more accurate than recognition of unfamiliar faces (Bruce, 1982; Bruce et al., 1999; Ellis et al., 1979; Klatzky & Forrest, 1984; Stacey et al., 2005). After two fixations, we are already able to evaluate familiarity (Van Belle et al., 2010) with the first fixation being located slightly above the tip of the nose (Bindemann et al., 2009; de Xivry et al., 2008; Hsiao & Cottrell, 2008). The second fixation is usually located on the left side of the face, especially in the area between eye and nose regardless of familiarity; all other fixations are mainly located in the eyes’ area (Van Belle et al., 2010). For a holistic face perception, the upper area of the face is the most important. Barton et al. (2006) observed more eye fixations in unfamiliar and morphed faces, while for familiar faces less upper-face scanning was found. They interpreted these gaze patterns as a mechanism to resolve ambiguity in morphed faces. For face recognition and identification, the upper face area is especially diagnostic. Prosopagnosia, for example, was shown to lead to less upper-face scanning and worse face recognition ability; the mouth was attended to most in familiar faces (de Xivry et al., 2008).

Evidence on how we perceive unfamiliar faces link the idea of psychological mechanisms promoting a fast detection of similarity to the self-face and functional perspectives on social gaze behavior. A striking example of this link is the fact that we fixate the eyes of other-race faces more frequently than those of our own race (Fu et al., 2012; Wang et al., 2013; Wheeler et al., 2011). Furthermore, there is evidence for an in-group homogeneity effect in same race faces (Wilson & Hugenberg, 2010). One plausible explanation for this evidence is that increased focus on the eye region reflects the intention to recognize others’ mental states (Emery, 2000; Grossmann, 2017), which might be especially necessary when confronted with faces of strangers (Hackel et al., 2014). The more someone looks like us, the less skeptical we are, and the less we make eye contact to reveal their mental state as we assume them to have a similar mental state as ourselves. Thus, similarity to our self-face leads to more trust and less eye gazing, as mental states and intentions are assumed similar (Baron-Cohen, 1997; DeBruine, 2002; Kobayashi & Kohshima, 1997 ). Many studies proved the eye region an important region of interest in human face perception; this is mainly due to the social value of the eye region (Birmingham et al., 2008; Grossmann, 2017; Itier et al., 2007; Senju & Johnson, 2009; Williams & Henderson, 2007; Yarbus, 1967; Young et al., 2014). Three types of information can be obtained through this area: intentions, emotions, and a person's gaze direction (Baron-Cohen, 1997; Baron-Cohen et al., 1997; Emery, 2000).

As similarity to the self-face seems to play an important role during face processing and perception, the stepwise transformation from other faces to the self-face is the main object of analysis in this article. Furthermore, in light of the differences between the processing of familiar and unfamiliar faces, this article will also be focused on face perception as a function of familiarity. No study to date shed light on how a strangers’ similarity to our own face influences our gaze patterns. More specifically, existing results highlight the importance of eye-directed gaze in social encounters (Emery, 2000; Grossmann, 2017; Risko et al., 2016) and it is this exact region, which we perceive differently depending on whether someone resembles us or not. Some studies found no differences between the self-face and familiar faces concerning electrophysiological responses (Tanaka et al., 2006). Other studies investigating the functional account of self-face processing found the self-face to be special in comparison to unfamiliar and familiar faces (Campbell et al., 2020; Keyes et al., 2010; Platek & Kemp, 2009; Uddin et al., 2005), showing special activation patterns in EEG as well as functional magnetic resonance imaging studies.

Eye-tracking studies also prescribe a special role to the self-face (Devue et al., 2009; Finke et al., 2017; Li & Tottenham, 2011).

Although findings are contradictory, we hypothesized that (1) as a function of similarity, the eyes’ area of those faces low in similarity is fixated more often and for a longer duration than the eyes’ area of highly similar faces. This effect will be independent of familiarity. As unfamiliar and morphed faces lead to more eye fixations (Barton et al., 2006) than familiar faces, we argue that this effect might be confounded when manipulating facial resemblance through morphing technique. Furthermore, we hypothesize that (2) subject’s similarity ratings are going to be faster for familiar faces than unfamiliar faces. Face recognition was shown to be faster in familiar than in unfamiliar faces (Bruce, 1982; Bruce et al., 1999; Ellis et al., 1979; Klatzky & Forrest, 1984; Stacey et al., 2005). For highly ambiguous stimuli (e.g., 50% morphs), we would await more fixations in the eye region as well as slower reaction times during similarity rating as ambiguity is higher (Barton et al., 2006). To test these hypotheses, we individually created a set of facial stimuli for each subject that systematically varied in terms of similarity to the self-face and familiarity by morphing their face into several other faces of the same sex including a close friend using advanced morphing technique.

We then recorded eye movement patterns while subjects were scanning those face stimuli analyzing individual regions of interest. Beyond the eye-tracking paradigm, we further applied a similarity-rating task to check how fast participants were able to judge similarity to their self-face of morphed faces. Although a body of research highlights how familiarity influences face perception, research on similarity is scarce. Thus, our study investigates how similarity to the self-face shapes eye-movement patterns while perceiving different faces.

## Material and Methods

### Participants

A total of 30 adult volunteers (18 females) participated in the study. The mean age of the study sample was 22.4 years (range: 19–27; *SD* = 1.96). The main requirement to be part of the study was to bring a close friend of the same sex (acquaintance-duration: minimum 1 month, with at least three times per week contact). Each of the participants had normal or corrected vision. Subjects with stable contact lenses were asked to wear glasses instead.

### Stimuli

Four different face identities served as individual stimuli: (a) the self-face, (b) one familiar face, and (c) two unfamiliar faces of the same sex. As (a) self-face a frontal portrait picture of each subject was taken, which was approved as suitable by the subject itself. For each subject-pair (a) self-faces were also used as (b) familiar faces for the group-mate, and as (c) unfamiliar faces, two moderately attractive faces of the same sex from the Regensburger picture set (Gründl, 2013) were chosen. All photos were taken without glasses, jewelry, or other objects; subjects were requested to show a neutral face. A mask that matched the average of all faces was modeled, which every face was brought into line with, so that the eyes, nose, and mouth would be in the same place. At the same time, the faces were separated from neck, ears, and hair. The same mask was used for all face-stimuli. Afterwards, the edges of the pictures were retouched with Adobe Photoshop CS6 so that no sharp edges or unnatural transitions would be visible; furthermore, all pictures were scaled to a size of 658 × 872 pixels. With the same software, a blue-, green-, and brown-eyed version of the unfamiliar faces were created which were matched with the subject’s eye color accordingly. All pictures were adjusted regarding root mean square (RMS) contrast (0.14) and luminance (0.50) in the red green blue (RGB) color space; finally, all pictures were set to black and white. The self-face was mirrored. The grey frame around the pictures was adjusted to the calculated luminance of all the pictures. The next step was to morph the four faces—(1) self-face, (2) familiar face, (3) unfamiliar face ×1, and (4) unfamiliar face ×2—into one another using “Abrosoft Fantamorph 5.4.5”; to morph the faces, at least 35 different points in from both faces were taken into account for creating the morph.

**Figure 1.**
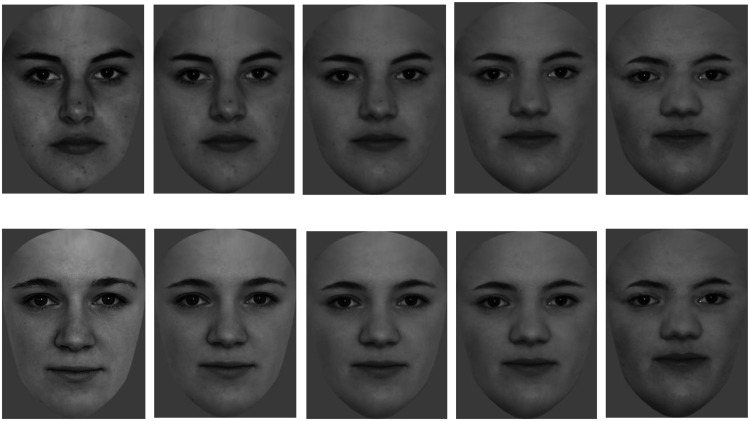
The transformation from unfamiliar/familiar face to the self-face.

For each subject, a gradual transformation from those other three faces to the self-face was created (self—familiar, self—unfamiliar ×1, and self—unfamiliar ×2) so that three sets containing five pictures with merging faces (25% steps between each picture) were developed. In sum, there were 15 different pictures, which served as stimuli for each subject (5 Morphs × 3 Faces). Special attention in this experiment was drawn to the 50% faces (50% self-face, 50% other face; see Figure 1), which offered a sophisticated classification of the dimensions familiarity and similarity to the self-face. The two unfamiliar faces were summarized to the category “unfamiliar,” as fixation count and fixation duration within the predefined areas of interest did not differ significantly between those two face stimuli; the 50% mix-faces were summarized to the categories “unfamiliar-morph” and “familiar-morph.”

**Figure 2.**
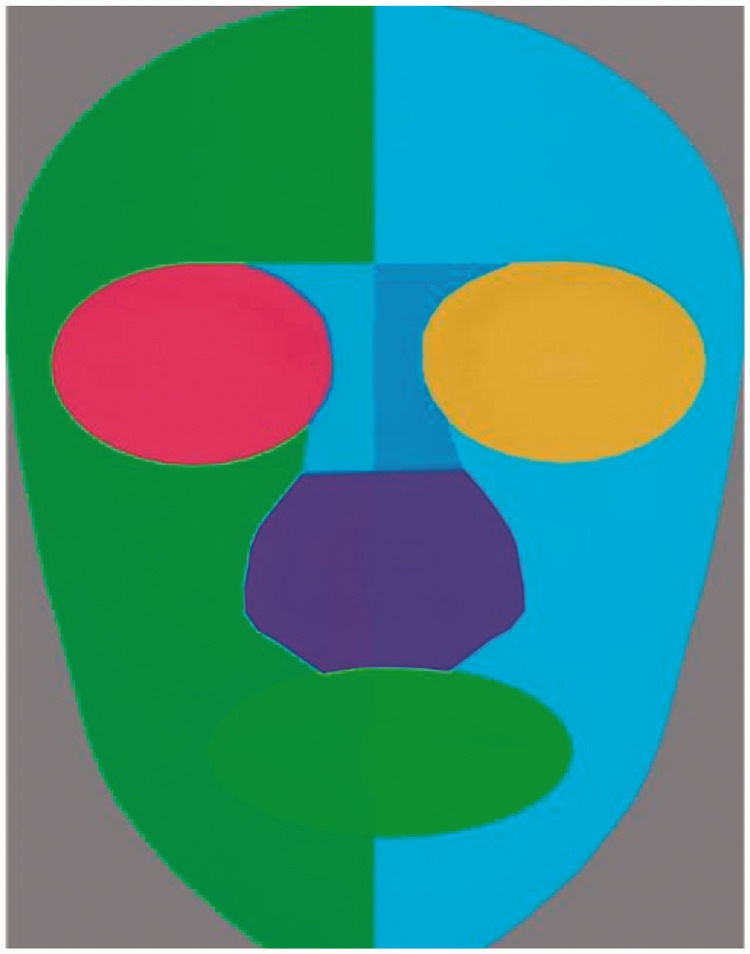
The predefined areas of interest (right eye, left eye, upper nose, lower nose, and mouth).

Finally, individual areas of interest (AOI) were defined for each of the generated faces with the help of “Tobii Pro Studio” (see Figure 2); this allows a precise tracking of the eye movement in those specific areas of the face, namely, the eyes, mouth, and nose area. The eyes’ area was calculated as a sum score of left and right eyes; the nose area was calculated as a sum score of upper and lower nose. Two parameters were calculated in those specified areas of interest: total fixation count and total fixation duration (across all trials).

### Tasks and Procedure

The experiment was conducted in two sessions: During the first session, subjects got their portrait pictures taken. During the second session, the eye-tracking task was performed where subjects had to view their individually created picture set; afterwards, subjects had to rate the same pictures in terms of similarity to their self-face. During the eye-tracking tasks, subjects were unaware of the similarity manipulation in order to prevent their gaze behavior to be biased. The second session was carried out 2 weeks after the first one.

### Eye-Tracking Task

The eye-tracking task was presented on a standard desktop computer (distance to screen: 60 cm; resolution: 1,920 × 1,080; refresh rate: 60 Hz; Dell Precision T-5610). A Tobii TX-300 eye-tracker (gaze-sampling frequency = 300 Hz, gaze sampling variability < 0.3%; accuracy = 0.4°, precision = 0.14°) was applied for video eye-tracking. Subjects were presented with the picture set in four randomized runs. Pictures were presented 3 seconds each with an interstimulus interval of 4 seconds (60 trials, 7 seconds per trial, total duration: ∼10 minutes.). All of the subjects were asked to sit straight in front of the monitor without crossing arms or legs. After a 9-point calibration of the eye-tracker, subjects were verbally instructed to relax, ideally not to move and to just look at the faces. Moreover, a written instruction on the display told them: “Now you are going to see a set of faces. Just have a look at them; you do not have to take action.”

### Similarity Rating Task

For the rating task, subjects were positioned in front of a second monitor (resolution: 1920 × 1080) where they were presented with the same picture set using E-Prime software. All of the final 15 pictures were presented in a randomized order twice (30 trials, 1.5 seconds duration, 1.5 seconds interstimulus interval, 3 seconds per trial, total duration: ∼ 5 minutes). After presentation, the image was replaced with a rating scale and subjects had to rate perceived similarity to their self-face on this scale ranging from one to nine (1 = *not similar* and 9 = *similar*) by pressing one of the numbers across the top of the keyboard; subjects were instructed to decide as quickly as possible and reaction times were assessed additionally. The distance from subject to display was not kept stable, so the visual angle is only an estimation (distance to display ∼ 60 cm); stimulus size in both tasks was about 17° × 23° of visual angle.

### Statistical Analysis

For correcting eye-tracking indices for AOI size, we multiplied each index by their respective AOI size and divided the result by the sum size of all three AOIs. With these new size-corrected indices, we computed all analyses to prove their robustness beyond AOI size (eyes: 61.65px, nose: 38.39px, mouth: 58.07px; sum size of all AOIs: 158.12px). For analysis of the eye-tracking indices, two repeated measures analysis of variance (ANOVA) models with fixation count and total fixation duration (in seconds) as dependent variables and similarity to the self-face (four morphing stages) as well as familiarity (unfamiliar vs. familiar) as within-subjects factors were conducted. In case of significant effects of similarity, post doc *t* tests were performed comparing individual morphing stages with one another (Bonferroni–Holm corrected *p* values for multiple testing). For the rating task, two repeated measures ANOVA models were computed with task index (similarity ratings and reaction times) as dependent variable and similarity as well as familiarity as within-subjects factors. Sphericity was tested using Mauchly’s test and in case of deviance from sphericity, Type I error was controlled by adjusting the degrees of freedom using the Greenhouse–Geisser correction. Partial eta squared and Cohen’s indicate effect sizes. All reported *p* values are two-tailed. Alpha levels were set at .05. Results are reported with original *df* and corrected *p* values. Data were analyzed using SPSS (version 25).

## Results

Sensitivity power analyses with G*Power (Faul et al., 2009) showed that our sample size of *N* = 30 was sufficient to detect a medium-sized effect of *f* = 0.25 with a statistical power of 1−β = .95 and α = .05 in all of the computed repeated measures ANOVA models (within subjects). Sample size was determined before any data analysis.

### Areas of Interest

Before analyzing the eye-tracking data for similarity and familiarity effects, differences between the three different AOI for total fixation count and total fixation duration were analyzed.

A repeated measures ANOVA model for total fixation count with familiarity (unfamiliar vs. familiar), AOI (eyes vs. nose vs. mouth), and morphing stage as within-subjects factors yielded a significant main effect for AOI, *F*(2, 58) = 117.29, *p* < .001, $\eta_{p}^{2}$ = .94. Significant differences between the eyes’ area and the nose, *F*(1, 29) = 39.78, *p* < .001, $\eta_{p}^{2}$ = 0.58, and mouth area, F(1, 29) = 229.11, *p* < .001, $\eta_{p}^{2}$ = 0.89, could be obtained. The eyes’ area got more fixations (*M* = 8.21) than the nose (*M* = 4.47) and mouth area (*M* = 1.06).

The same model was computed for total fixation duration; again, a significant main effect for AOI, *F*(2, 58) = 124.35, *p* <. 001, $\eta_{p}^{2}$ = .81, was found. Significant differences between the eyes’ area and the nose, *F*(1, 29) = 100.24, *p* < .001, $\eta_{p}^{2}$ = 0.78, and mouth area *F*(1, 29) = 168.60, *p* < .001, $\eta_{p}^{2}$ = 0.85, could be obtained. The eyes’ area was fixated longer (*M* = 2.15) than the nose (*M* = 0.71) and mouth area (*M* = 0.39). This result highlights the importance of the eyes’ area in this investigation. The following analyses were thus performed with indices of the eyes’ area only.

### Eye-Tracking Indices

**Figure 3.**
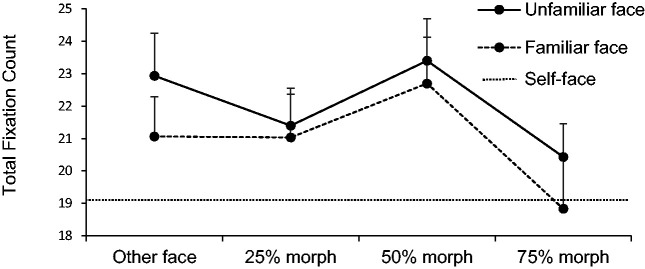
Total fixation count of the eyes’ area of the two different morphing stimuli, ranging from unfamiliar and familiar face to the 75% morph (stepwise transformation). The self-face is included as a baseline. Error bars represent 95% within-subject errors.

For total fixation count, the repeated measures ANOVA model yielded a strong main effect for familiarity, *F*(1, 29) = 6.59, *p* = .02, $\eta_{p}^{2}$ = 0.19, as well as similarity, *F*(3, 87) = 7,68, *p* < .01, $\eta_{p}^{2}$ = 0.21; see Figure 3. The interaction did not reach significance, *F*(3, 87) = 0.44, *p* = .73, $\eta_{p}^{2}$ = 0.02. Post hoc *t* tests revealed significant differences between the other face and the 75% morph, *t*(29) = 3.22, *p* < .01, *d* = 0.59, as well as between the 50% and 75% morph, *t*(29) = 4.65, *p* < .001, *d* = 0.85. No significant difference was obtained between the other face and the 50% morph, *t*(29) = −1.43, *p* = .31.

For comparing each morphing stage of the unfamiliar face to the baseline (self-face), paired samples *t* tests were performed, which yielded significant differences between the first three morphing stages and the self-face (all *t*(29)> 2.97, all *p* < .01, all *d* > .54). For the transition from familiar to the self-face, only the familiar face and the 50% morph differed significantly from the self-face (both *t*(29) > 2.24, *p* < .033, both *d* > 0.41).

The repeated measures ANOVA for total fixation duration model also revealed strong main effects for familiarity, *F*(1, 29) = 7.36, *p* = .01, $\eta_{p}^{2}$ = 0.20, as well as similarity

**Figure 4.**
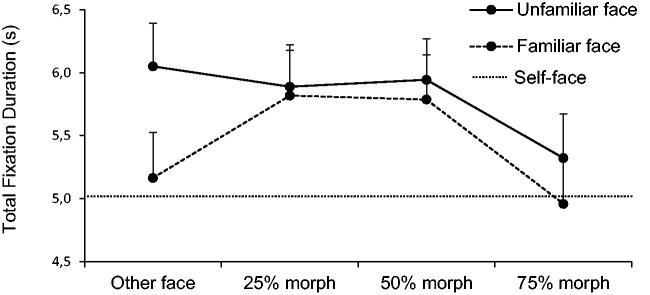
Total fixation duration (in seconds) of the eyes’ area of the two different morphing stimuli, ranging from unfamiliar and familiar face to the 75% morph (stepwise transformation). The self-face is included as a baseline. Error bars represent 95% within-subject errors.

*F*(3, 87) = 4.98, *p* < .01, $\eta_{p}^{2}$ = 0.15; see Figure 4). No significant interaction was found *F*(3, 87) = 1.89, *p* = .14, $\eta_{p}^{2}$ = 0.06. Post hoc *t* tests revealed significant differences between the 25% and 75% morph, *t*(29) = 2.99, *p* = .03, *d* = 0.55, and between the 50% and 75% morph, *t*(29) = 3.88, *p* < .01, *d* = 0.71. No significant difference was obtained between the other face and the 50% morph, *t*(29) = −1.34, *p* = .57.

Paired samples *t* tests were performed for comparing each morphing stage of the unfamiliar stimuli to the self-face showing again significant differences between the first three stages and the self-face (all *t*(29) > 4.00, all *p* < .01, all *d* > 0.73). For familiar stimuli, only the 25% and 50% morph differed significantly from the self-face (all *t*(29) > 3.16, all *p* < .01, all *d* > 0.58).

### Perceived Similarity

**Figure 5.**
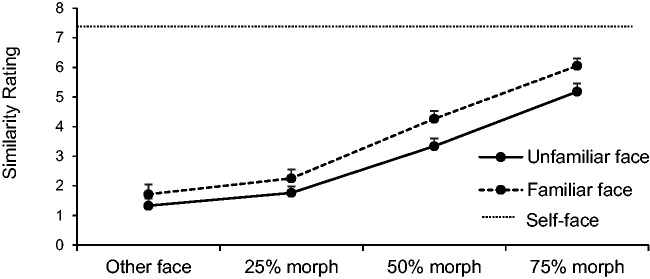
Similarity ratings of the two different stimuli across morphing stages. Error bars represent 95% within-subject errors.

To test whether similarity ratings of the morphed stimuli were accurate for both unfamiliar and familiar faces, a repeated measures ANOVA model was performed, which yielded a strong main effect for similarity, *F*(3, 87) = 247.56, *p* < .01, $\eta_{p}^{2}$ = 0.90, as well as for familiarity *F*(1, 29) = 37.37, *p* < .01, $\eta_{p}^{2}$ = 0.56; see Figure 5. The interaction did not reach significance *F*(3, 87) = 2.31 *p* = .08, $\eta_{p}^{2}$ = 0.07. Post hoc *t* tests yielded significant differences between the other face and all three morphing stages (all *t*(29) > 6.31, all *p* < .01, all *d* > 1.15). Familiar and unfamiliar stimuli differed significantly from each other, *t*(29) = 6.11 *p* < .01, *d* = 1.12.

**Figure 6.**
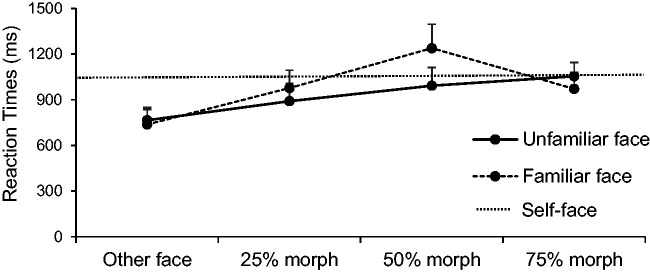
Reaction times of the similarity ratings of the two different stimuli across morphing stages. Error bars represent 95% within-subject errors.

The same model was performed for reaction times of the rating task; it yielded a significant main effect of similarity, *F*(3, 87) = 12.52, *p* < .01, $\eta_{p}^{2}$ = 0.30; neither the main effect for familiarity nor the interaction reached significance. Figure 6 shows the reaction times of those ratings additionally; post hoc *t* tests yielded significant differences between the other face and all three morphing stages (all *t*(29) > 3.69, all *p* < .01, all *d* > 0.67). No significant difference between familiar and unfamiliar stimuli was found, *t*(29) = 1.13, *p* = .27.

## Discussion

Our study aimed to investigate face recognition under the condition of modulated similarity to the self-face. Findings provide evidence that similarity to the self-face shapes the way we look at the eyes of others. Supporting our first hypothesis, the eye region of faces low in similarity received more and longer fixations than those of highly similar ones. Nevertheless, familiarity also had a significant influence on eye fixations as unfamiliar faces got more and longer fixations than familiar ones. No interaction was found, though. Most importantly, our results show that individuals fixate the eyes’ region of dissimilar strangers longer and more often than that of similar ones. Thus, similarity might be another important factor to explain variance within gaze patterns besides familiarity. Furthermore, our findings resemble earlier evidence showing more eye fixations in morphed stimuli (Barton et al., 2006). This pattern was interpreted as a mechanism to resolve ambiguity. Moreover, subjects rated similarity to their self-face accurately. However, reaction times were faster for dissimilar stimuli. This result stands in contrast to the notion that self-face recognition is faster and more accurate than recognition of familiar or unfamiliar faces (Keenan et al., 2000). Recognition of unfamiliar faces is usually weaker and less stable (Bruce et al., 1999; Rossion et al., 2003). Our results might therefore be an indicator that face processing may not be as easily explained by familiarity, as it seems.

The fact that the eyes of the self-face got significantly fewer and shorter fixations than unfamiliar ones is in line with our hypothesis as well as with results of other studies (Althoff & Cohen, 1999; Barton et al., 2006, Heisz & Shore, 2008). Moreover, the 75% morphs got less and shorter fixations than unfamiliar or familiar faces. The 25% and 50% morphs, on the other hand, got a similar amount and duration of fixations as the stranger faces. For an accurate judgment of their intentions, it is presumably necessary to fixate the eyes’ area of those morphed faces more often and longer (Baron-Cohen, 1997; Kobayashi & Kohshima, 1997). As they are unfamiliar but also partly similar to the viewer, they are also more complex and ambiguous to perceive (Barton et al., 2006). Thus, our results highlight how we look at very dissimilar strangers in comparison to more similar ones, underlining the effect of less eye contact in those individuals resembling our own face. This pattern was similar both for unfamiliar and familiar face stimuli, indicating that familiarity plays a minor role here.

As morphed faces progressed higher in similarity to the self-face, the eyes’ area got less and less important for perceiving the face as similar to the self-face; thus, fixations toward the eyes’ area decreased in number and duration. As eye-directed gazing is especially important for social interactions, one might argue that in individuals resembling ourselves, eye-directed gaze is less important as we prescribe similar intentions and mental states to them (Baron-Cohen, 1997; Kobayashi & Kohshima, 1997). Thus, we argue that increased gazing toward the eyes of dissimilar people might reflect the need to read the intentions of a distrusted stranger (Baron-Cohen, 1997; DeBruine, 2002; Kobayashi & Kohsima, 1997). Attention to the eyes also serves as a tool for social communication (Gobel et al., 2015; Risko et al., 2016; Wu et al., 2014). Earlier findings suggested that the self-face draws more attention and is thus more difficult to avoid when presented within a crowd of faces (Devue et al., 2009). Furthermore, morphed faces receive more fixations of the eyes’ area as ambiguity needed to be resolved (Barton et al., 2006). Nevertheless, it is a completely different approach to let subjects search or recognize certain faces; gaze patterns might change with the task goal. In our task, subjects just had to look at the faces without recognizing them. There were no confounding other faces, as only one face per trial was presented. On the other hand, Campbell et al. (2020) found an increased electrophysiological response to the self-face compared with familiar and unfamiliar faces. This fact again highlights the special processing of the self-face; subjects were requested to identify faces here, which might modulate the physiological response in comparison to a free viewing paradigm.

It is worth noticing that eye-directed gaze does not only serve as an information gathering but also as a potent social signal (Grossmann, 2017). Convincing evidence supports the notion that social gaze behavior not merely acts in the service of information gathering about our sensory environment but rather as a tool for social communication (Maran et al., 2019; Risko et al., 2016). For example, when we watch a group on a video tape we tend to look more toward individuals resembling high status; this pattern reverses when those individuals can see where we are looking at (Gobel et al., 2015). Taking this social signaling function of eye-directed gaze into account, our findings reflect an attempt to signal strangers that they are being watched. This might be functional as being watched does lead to prosocial behaviors, which is desirable when we meet strangers. Being watched does indeed enhance cooperation (Bateson et al., 2006) and prosocial behavior (Nettle et al., 2013; Pfattheicher & Keller, 2015). It is worth noticing that facial similarity also serves as a key factor enhancing cooperation among humans (Fischer, 2009). Therefore, it might be plausible that from a social signaling perspective of gaze behavior, eye-directed gaze toward dissimilarly looking strangers might be instrumental to shape their readiness to act prosocial or at least to reduce harmful intentions.

As subjects rated similarity to their self-face faster for dissimilar stimuli than for their self-face, we assume that this effect might be due to visual adaptation to the morphed face stimuli used in both the eye-tracking and rating paradigm. Rooney et al. (2012) demonstrated that familiar faces are subject to rapid effects of adaptation, which means that being exposed to unfamiliar distorted face stimuli eminently influences the recognition of other familiar faces. In demonstrating this effect, they were able to prove that cross-identity adaptation takes place, from unfamiliar to familiar faces. Obviously, the method in our experiment is different from other studies investigating face perception processes, as subjects were confronted with morphed stimuli containing their self-face. Nevertheless, the gradual transformation from other faces to the self-face might lead to slower self-face recognition, as the contrast between self and other faces is easier to detect than the difference between a morph and the self-face. Thus, we argue that subjects might have rapidly adapted to those morphed face stimuli, which influences their self-face recognition. Furthermore, in our natural environment, we usually process other faces more often than our own face. Finally yet importantly, there is no evidence that self- and other-face perception are processed separately on a neural level (Rooney et al., 2012). Thus, one might conclude that there is no level of self-other distinction but a level of facial identity, which influences our face perception. As we found no significant difference between familiar and unfamiliar faces concerning RTs in the rating paradigm, it seems likely that adaptation to morphed stimuli took place here.

### Limitations and Future Research Directions

Some authors argued that using sum scores for fixation count or duration might be too vague to detect differences between different kinds of stimuli; they only found differences by comparing fixations separately (Van Belle et al., 2010). Thus, the dynamics of gaze patterns over time would be valuable to analyze. It would also be interesting to manipulate familiarity experimentally by morphing a familiar face into an unfamiliar face. To increase the ecological validity of eye-tracking, it would also be useful to apply mobile eye-tracking systems such as glasses to examine naturalistic eye movement patterns during face recognition and processing. Our results show that gaze behavior differs significantly between different kinds of facial stimuli, while similarity to the self-face serves as another key factor explaining gaze patterns alongside familiarity. Future studies should investigate whether similarity to the self-face might explain the other race effect better than the simple categorization of races. Research, for example, implies that race and emotion cues are processed parallel, which might also hold true for similarity (Kubota & Ito, 2007).

## Conclusion

When you think back to the situation in the shopping mall, where you were confronted with a stranger. How would you react? Our study suggests that you look a stranger less in the eyes if he or she resembles you. Gazing toward the eyes’ area reflects the value of similarity to the self-face as a distinct factor during face perception. Our study is the first to link similarity to the self-face to social gaze patterns, more specifically gazing toward the eyes of others.
